# Binding of ATP at the active site of human pancreatic glucokinase – nucleotide-induced conformational changes with possible implications for its kinetic cooperativity

**DOI:** 10.1111/j.1742-4658.2011.08160.x

**Published:** 2011-05-31

**Authors:** Janne Molnes, Knut Teigen, Ingvild Aukrust, Lise Bjørkhaug, Oddmund Søvik, Torgeir Flatmark, Pål Rasmus Njølstad

**Affiliations:** 1 Department of Pediatrics, Haukeland University HospitalBergen, Norway; 2 Department of Clinical Medicine, University of BergenNorway; 3 Department of Biomedicine, University of BergenNorway; 4 Center for Medical Genetics and Molecular Medicine, Haukeland University HospitalNorway

**Keywords:** ATP binding, catalytic mechanism, *GCK* maturity onset diabetes of the young (*GCK*-MODY), glucokinase, kinetic cooperativity

## Abstract

Glucokinase (GK) is the central player in glucose-stimulated insulin release from pancreatic β-cells, and catalytic activation by α-d-glucose binding has a key regulatory function. Whereas the mechanism of this activation is well understood, on the basis of crystal structures of human GK, there are no similar structural data on ATP binding to the ligand-free enzyme and how it affects its conformation. Here, we report on a conformational change induced by the binding of adenine nucleotides to human pancreatic GK, as determined by intrinsic tryptophan fluorescence, using the catalytically inactive mutant form T228M to correct for the inner filter effect. Adenosine-5′-(β,γ-imido)triphosphate and ATP bind to the wild-type enzyme with apparent [L]_0.5_ (ligand concentration at half-maximal effect) values of 0.27 ± 0.02 mm and 0.78 ± 0.14 mm, respectively. The change in protein conformation was further supported by ATP inhibition of the binding of the fluorescent probe 8-anilino-1-naphthalenesulfonate and limited proteolysis by trypsin, and by molecular dynamic simulations. The simulations provide a first insight into the dynamics of the binary complex with ATP, including motion of the flexible surface/active site loop and partial closure of the active site cleft. In the complex, the adenosine moiety is packed between two α-helices and stabilized by hydrogen bonds (with Thr228, Thr332, and Ser336) and hydrophobic interactions (with Val412 and Leu415). Combined with enzyme kinetic analyses, our data indicate that the ATP-induced changes in protein conformation may have implications for the kinetic cooperativity of the enzyme.

## Introduction

Glucokinase (GK) or hexokinase IV (EC2.7.1.1) catalyses the phosphorylation of α-d-glucose (Glc) to form glucose 6-phosphate, the entry point of Glc into glycolysis, using MgATP^2−^ as the phosphoryl donor. Human GK (hGK) is expressed in the liver [1], pancreas [2], brain, and endocrine cells of the gut [3,4]. It is a key regulatory enzyme in the human pancreatic β-cell (isoform 1), playing a crucial role in the regulation of insulin secretion, and is therefore termed the pancreatic β-cell glucose sensor [5]. In humans, more than 600 different mutations in the glucokinase gene (*GCK*) have been detected in patients suffering from familial, mild fasting hyperglycaemia [*GCK* maturity onset diabetes of the young (*GCK*-MODY), *GCK* permanent neonatal diabetes mellitus, and *GCK* congenital hyperinsulinism of infancy [6–11]. Some of the mutations greatly reduce the binding affinity of MgATP^2−^ [11,12], which is compatible with a direct interaction of these residues with the nucleotide at the active site.

The catalytic mechanism of GK has been the subject of several detailed analyses, and is still a partly unresolved issue. Although some theoretical evidence has been presented in support of a random order mechanism, in which the enzyme interacts with the substrate and cosubstrate in a random fashion [13], enzyme kinetic studies support an ordered mechanism in which Glc binds to the enzyme before the cosubstrate [14–16]. The discussion is reminiscent of that related to the catalytic mechanism of yeast hexokinase [17]. For both enzymes, part of the discussion has been related to the question of whether ATP binds to the Glc-free enzyme and the possibility of a nucleotide-triggered change in protein conformation.

In this work, we have studied the interaction of ATP and analogues with the human pancreatic enzyme with the aims of: (a) presenting experimental evidence for equilibrium binding to the ligand-free super-open conformation; (b) demonstrating possible conformational changes associated with ATP binding; (c) obtaining insights into the active site contact residues involved in ATP binding; and (d) relating this information to steady-state enzyme kinetic data. To achieve these aims, we used a combined experimental approach including intrinsic tryptophan fluorescence (ITF), extrinsic 8-anilino-1-naphthalenesulfonate (ANS) fluorescence, limited proteolysis, and molecular dynamic (MD) simulations. Additionally, enzyme kinetic analyses were performed to evaluate the functional implications of the structural data. The different approaches provide new insights into the interaction of ATP with hGK, with possible implications for the positive kinetic cooperativity with respect to Glc.

## Results

### Recombinant proteins

The average yields of soluble recombinant pancreatic glutathione-*S*-transferase (GST)–hGK fusion proteins were ∼ 4.0 mg L^−1^ (wild type and T228M) and ∼ 2.0 mg L^−1^ (L146R). As the recombinant wild-type (WT) hGK and WT GST–hGK enzymes demonstrate similar steady-state kinetic parameters and the same apparent *K*_d_ for Glc in the ITF equilibrium binding assay [18], the fusion proteins were used in kinetic studies and ITF equilibrium binding analyses with Glc. In the adenine nucleotide (AdN) equilibrium binding studies, we compared nontagged and GST-tagged GK. In all other experiments, only the nontagged proteins were used.

### Characterization of the T228M mutant reference enzyme

**Figure 1:**
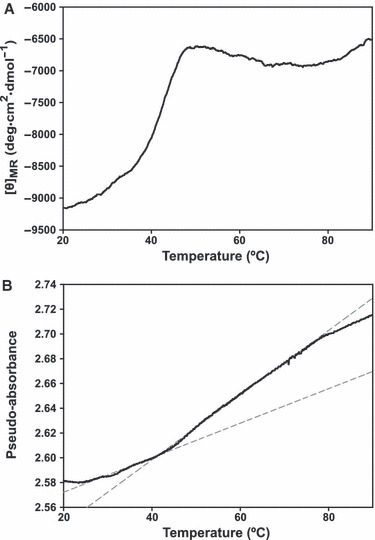
Thermal refolding–unfolding and aggregation of WT hGK. The experiments were performed as described in Experimental procedures. (A) The thermal refolding–unfolding profile of WT hGK (23 μm) in the absence of Glc was determined by following the change in ellipticity at 222 nm at a constant heating rate of 40 °C·h^−1^. An apparent transition temperature (*T*_m_) of 42.4 ± 0.2 °C was determined from the first derivative of the smoothed denaturation curve. No significant difference in the profile was observed in the presence of Glc (data not shown). The observed optical activity is expressed as the mean residue molar ellipticity ([*θ*]_MR_). (B) The pseudo-absorbance data were obtained at the same time as the CD data in (A), reporting on the biphasic heat-induced increase in absorbance. The regression lines, based on data points in the temperature interval 24–79 °C, indicate an inflection point at ∼ 42 °C and increasing aggregation of the protein above this temperature; above ∼ 80 °C, the absorbance decreased, probably owing to precipitation of the protein.

The T228M mutant form, causing *GCK*-MODY in the heterozygous state and *GCK* permanent neonatal diabetes mellitus in the homozygous state [9,19], was selected as a non-ATP-binding reference enzyme on the basis of its previously described kinetic properties [9,20,21]. Here, equilibrium binding of Glc, as determined by ITF, demonstrated an increased affinity (*K*_d_ = 3.1 ± 0.1 mm) in comparison with WT GST–hGK (*K*_d_ = 4.3 ± 0.1 mm), and a fluorescence enhancement signal response [(Δ*F*_eq_/*F*_0_)_max_ × 100] similar to that of the wild type (Table 1). Steady-state kinetic analyses demonstrated a ∼ 9000-fold reduced catalytic activity (*k*_cat_ ∼ 7 × 10^−3^ s^−1^) (Table 1). Thr228 is a highly conserved residue at the active site of the hexokinase family of enzymes, positioned in the phosphate-binding loop and part of a classical ATP-binding motif (phosphate 2 site) in hexokinases and homologous proteins [22]. In the crystal structures of human and yeast hexokinases, the hydroxyl group of this conserved Thr interacts with the α-phosphate of ATP [21,23,24], and a Thr→Met substitution in hGK is inferred to eliminate this important contact (see the *in silico* studies below). According to the coordinates of the closed (Glc-bound) conformation of WT hGK [Protein Data Bank (PDB) ID 1v4s], the T228M mutation is predicted to be destabilizing, as measured by the free energy of thermal unfolding (ΔΔ*G* = −4.07 kcal·mol^−1^) and the free energy of folding (ΔΔ*G* = 0.85 kcal·mol^−1^). However, the far-UV CD spectrum was very similar, if not identical, to that of WT hGK (Fig. S1), and no significant differences in the apparent *T*_m_ values (on thermal unfolding) of WT hGK (Fig. 1) and the mutant protein (data not shown) were observed. Thus, the Thr→Met substitution has little impact on the protein fold.

**Table 1:** The steady-state kinetics and ITF properties of WT GST–hGK and two *GCK*-MODY mutant forms. NM, not measurable.

	WT	T228M^a,b^	L146R
*k*_cat_ (s^−1^)^c^	67.6 ± 1.3	7 × 10^−3^	0.77 ± 0.03
*k*_cat_ (s^−1^)^d^	68.4 ± 0.9	NM	0.61 ± 0.03
Relative catalytic activity (%)	100	0.01	1.0
[S]_0.5_ Glc (mm)	8.23 ± 0.26	NM	352 ± 25
*K*_m_ MgATP^2−^ (mm)	0.16 ± 0.01	NM	0.24 ± 0.04
Hill coefficient (*n*_H_)^c^	1.95 ± 0.19	NM	1.29 ± 0.04
Hill coefficient (*n*_H_)^d^	1.15 ± 0.04	NM	0.73 ± 0.04
Glc response (%) [(Δ*F*_eq_/*F*_o_)_max_ × 100]	28.7 ± 1.5	29.2 ± 0.1	5.3 ± 0.5
*K*_d_ Glc (mm)^e^	4.3 ± 0.1	3.1 ± 0.1	19.3 ± 3.8

a The *n*_H_, [S]_0.5_ and *K*_d_ values were not measured, because of low catalytic activity. ^b^ The ITF responses to 200 mm Glc were 33.2 and 36.0 arbitrary fluorescence units for the fusion protein and the isolated T228M hGK mutant, respectively. ^c^ Assay with Glc as the variable substrate. ^d^ Assay with ATP as the variable substrate. ^e^ Obtained from equilibrium binding measurements by intrinsic Trp fluorescence spectroscopy.

### Equilibrium binding of adenosine-5′-(β,γ-imido)triphosphate (AMP-PNP), ATP and MgATP to the ligand-free enzyme

**Figure 2:**
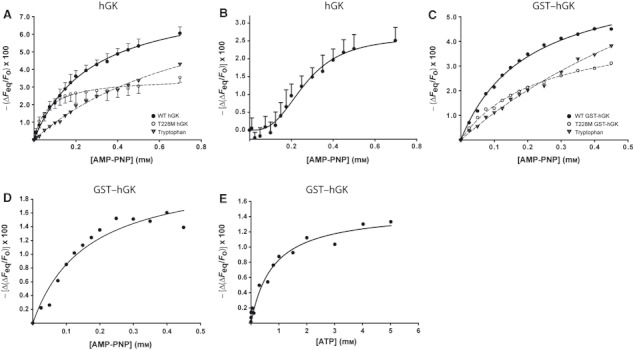
Equilibrium binding of AMP-PNP (A–D) and ATP (E) in the absence of Glc. (A) The change in fluorescence intensity [(Δ*F*_eq_/*F*_0_) × 100] was measured at 25 °C upon subsequent additions of ligand. (A) AMP-PNP titration curves of WT hGK (•), the non-ATP-binding mutant T228M hGK (○), and free Trp (at a concentration giving the same *F*_0_ value as the enzyme) (▾). The data were fitted to binding isotherms by nonlinear regression analysis, with *r*^2^ > 0.99 for both WT hGK and T228M hGK. Data points and error bars represent the mean ± SD of three independent titrations. (B) The net fluorescence quenching [Δ(Δ*F*_eq_/*F*_0_)_max_ × 100] of WT hGK as a function of [AMP-PNP], with a calculated [L]_0.5_ value of 0.27 ± 0.02 mm. The data points and the solid line represent the difference between the WT and T228M hGK titrations. (C) The same experiment as in (A), but performed on the GST fusion proteins. The titration curves of WT GST-hGK (•), the non-ATP-binding mutant T228M GST–hGK (○), and free Trp (at a concentration giving the same *F*_0_ value as the enzyme) (▾). The data were fitted to binding isotherms by nonlinear regression analysis, with *r*^2^ > 0.99 for both WT GST–hGK and T228M GST–hGK. (D) The net fluorescence quenching [Δ(Δ*F*_eq_/*F*_0_)_max_ × 100] of WT GST–hGK as a function of [AMP-PNP], with a calculated [L]_0.5_ value of 0.16 ± 0.04 mm. The data points and the solid line represent the difference between the WT and T228M GST–hGK titrations. (E) Equilibrium binding of ATP to WT GST–hGK in the absence of Glc. The figure shows the net decrease in ITF [Δ(Δ*F*_eq_/*F*_0_)_max_ × 100] with increasing concentrations of ATP (25 °C), calculated in a similar manner as in (B) and (D), representing the difference between the WT GST–hGK and T228M GST–hGK titrations. The data were fitted to a hyperbolic binding isotherm by nonlinear regression analysis (*r*^2^ > 0.97), and an [L]_0.5_ value for ATP of 0.78 ± 0.14 mm was calculated. The data points (•) represent the means of duplicate titration experiments.

To study binding of AdNs to the ligand-free nontagged enzyme, we first measured the change in ITF [(Δ*F*_eq_/*F*_0_) × 100] at 25 °C as a function of the AdN concentration. In contrast to the enhancement of the ITF signal observed with Glc [18,25], the ATP analogue AMP-PNP resulted in quenching of the fluorescence (Fig. 2), consistent with a previous report [26]. However, the inner filter effect resulting from nucleotide absorbance at the excitation wavelength (295 nm), which was not considered in that report, made a significant contribution to the quenching. To correct for this effect, a similar titration was performed with the non-ATP-binding mutant T228M and with free Trp (Fig. 2A,C). Of the two reference titrations, the T228M mutant gave the preferred correction (Fig. 2A), as the mutant also demonstrated quenching of the ITF at low concentrations (≤ 0.1 mm). From the fluorescence difference data (Fig. 2B), an apparent [L]_0.5_ (ligand concentration at half-maximal effect) value of 0.27 ± 0.02 mm (25 °C) was estimated by nonlinear regression analysis. The net (specific) fluorescence quenching observed for AMP-PNP was modest, but significant [Δ(Δ*F*_eq_/*F*_0_)_max_ × 100 = −2.6% ± 0.2%], suggesting that one or more of the three Trp residues (Trp99, Trp167, and Trp257) undergo small changes in quantum yield, but without any significant spectral shift. A similar result was obtained with the respective GST–hGK fusion proteins (Fig. 2C,D), with an [L]_0.5_ value of 0.16 ± 0.04 mm and Δ(Δ*F*_eq_/*F*_0_)_max_ × 100 = −2.2% ± 0.2%. In the ITF titrations of the wild type and the T228M mutant (control) with increasing concentrations of ATP (Fig. 2E), a net decrease in fluorescence intensity similar to the AMP-PNP response was observed. The differential binding data (Fig. 2E) were fitted to a hyperbolic binding isotherm by nonlinear regression (*r*^2^ > 0.97), giving a half-maximal effect ([L]_0.5_) at 0.78± 0.14 mm and Δ(Δ*F*_eq_/*F*_0_)_max_ × 100 = −1.5% ± 0.1%. Similar titrations with MgATP gave comparable maximal quenching of ITF of Δ(Δ*F*_eq_/*F*_0_)_max_ × 100= −2.2% ± 0.3%.

### Thermal refolding and unfolding

As previously demonstrated by ITF, ligand-free WT hGK senses temperature shifts from 4 to 39 °C directly by a slow (seconds to minutes) conformational change (hysteresis), with a biphasic time course in temperature jump (4–39 °C) experiments [18]. The far-UV CD spectroscopy at 222 nm confirmed this conformational change by an apparent change in the secondary structure in the same temperature range (Fig. 1A). At higher temperatures, the enzyme demonstrated relatively low global thermodynamic stability, with an apparent *T*_m_ of 42.4 ± 0.2 °C and increasing aggregation at temperatures ≥ 42 °C, as measured from the associated high-voltage (pseudo-absorbance) curve obtained at the same time (Fig. 1B). Similarly, the isothermal (25 °C) chemical unfolding caused by guanidine chloride also resulted in aggregation of the protein (data not shown). This instability of the protein precluded an estimate of equilibrium thermodynamic parameters, and thus also measurement of the effect of ligands on such conformational equilibria.

### Effect of ATP and Glc on extrinsic ANS fluorescence and limited proteolysis

**Figure 3:**
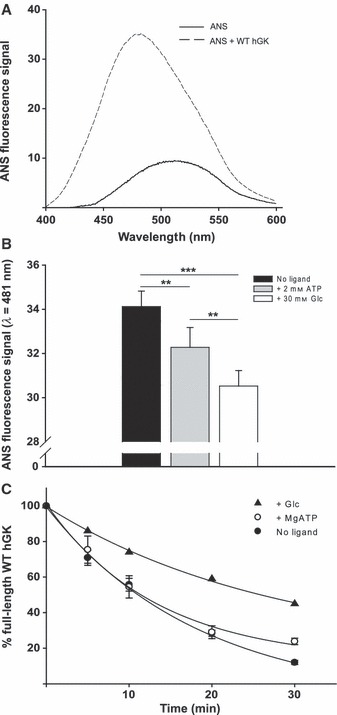
ANS fluorescence measurements and limited proteolysis. (A) Emission fluorescence spectra (*λ*_ex_ = 385 nm) of free ANS in buffer and ANS in the presence of 0.75 μm WT hGK. A final ANS concentration of 60 μm was used. (B) The effect of ATP and Glc on ANS binding to WT hGK. The ANS binding experiments were performed at a temperature of 38 °C, as described in Experimental procedures, with 60 μm ANS and a protein concentration of 0.75 μm. The concentrations of Glc and ATP were 30 mm and 2 mm, respectively. Each column represents the mean ± SD of three independent experiments. Statistical significance was determined with Student’s *t*-test: **P < 0.01 and ***P < 0.0001. (C) Time-course for the limited proteolysis of WT hGK by trypsin. WT GST–hGK (0.5 mg·mL^−1^) was cleaved with factor Xa for 2 h at 4 °C, and subsequently subjected to limited proteolysis by trypsin at 25 °C (trypsin/hGK ratio of 1 : 400 by mass) in the absence of ligand (•), or in the presence of either 40 mm Glc (▴) or 2 mm ATP/4 mm MgAc (○). Data points and error bars represent the mean ± SD of three independent experiments.

ANS is an extrinsic fluorophore with affinity for hydrophobic clusters in proteins that are not tightly packed in a fully folded structure or become exposed in partially unfolded structures [27]. The weak fluorescence of ANS was greatly enhanced upon binding to ligand-free WT hGK (Fig. 3A), with a maximum at ∼ 480 nm (blue shift), indicative of ANS binding to exposed hydrophobic clusters. As seen from Fig. 3B, both ATP and Glc significantly reduced the ANS fluorescence signal [Glc (*P* = 0.00004) > ATP (*P* = 0.004)], compatible with a decrease in accessible hydrophobic clusters as compared with the ligand-free enzyme.

In our studies on mutant forms of hGK, their susceptibilities to limited proteolysis by trypsin have proved to be a valuable conformational probe (unpublished data). Here, it was demonstrated (Fig. 3C) that the ligand-free WT hGK (at 25 °C) is partly stabilized by its association with ATP and Glc (Glc > ATP).

### Effect of nonhydrolysable ATP analogues on the equilibrium binding of Glc

**Figure 4:**
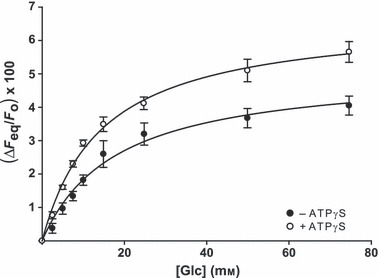
The Glc binding isotherm for the mutant L146R GST–hGK. The enhancement of ITF was measured at 25 °C with increasing concentrations of Glc in the absence (•) and presence (○) of 2.5 mm ATPγS. The solid lines represent the fit of the data to two hyperbolas as obtained by nonlinear regression analyses, giving *K*_d_ values of 19.3 ± 3.8 mm (*r* ^2^ > 0.98) and 14.0 ± 1.4 mm (*r* ^2^ > 0.99) in the absence and presence of ATPγS, respectively, and a fluorescence signal response [(Δ*F*_eq_/*F*_0_)_max_ × 100] of ∼ 5%. For comparison, the (Δ*F*_eq_/*F*_0_)_max_ × 100 was ∼ 30% for WT GST–hGK. Data points and error bars represent the mean ± SD of three independent experiments.

The equilibrium binding of Glc to the ligand-free WT hGK and its binary AdN complexes was determined by its enhancement of the ITF signal (Table 2). In the absence of AdNs, a hyperbolic binding isotherm for Glc was observed, with a *K*_d_ value of 4.2 ± 0.1 mm at 25 °C. Titration with Glc in the presence of Mg-adenosine-5′-*O*-(3-thiotriphosphate) (ATPγS) and MgAMP-PNP also gave hyperbolic binding isotherms; however, the apparent affinity for Glc increased (Table 2), i.e. about two-fold with 5 mm MgAMP-PNP (*P* = 0.002). A similar effect was observed for the *GCK*-MODY L146R mutant in the presence of 2.5 mm ATPγS; that is, the apparent *K*_d_ decreased from 19.3 ± 3.8 mm to 14.0 ± 1.4 mm (Fig. 4), and there was a ∼ 25% increase in the fluorescence signal response [(Δ*F*_eq_/*F*_0_)_max_ × 100]. The mutant demonstrated a ∼ 100-fold reduction in *k*_cat_ and a ∼ 40-fold increase in the [S]_0.5_ (substrate concentration at half-maximal activity) value for Glc (Table 1). The positive kinetic cooperativity with respect to Glc was partly lost in the mutant (*n*_H_ = 1.29 ± 0.04), and in contrast to previous findings [28], the *K*_m_ for ATP (0.24 ± 0.04 mm) was only slightly increased.

**Table 2:** The effect of ATP analogues on the equilibrium binding affinity of Glc as determined by ITF fluorescence titrations on WT GST–hGK.

Concentration (mm)	*K*_d_ (mm)
No ligand	4.2 ± 0.1^a^
MgAMP-PNP	
1	2.6 ± 0.1^b^
5	2.1 ± 0.1^c^
MgATPγS	
1	4.0 ± 0.1^b^
3	2.8 ± 0.1^b^

a Mean ± SD of five independent titration experiments. ^b^ Based on nonlinear regression analysis of single binding isotherms (*r* ^2^ > 0.99) (*n* = 12 data points). ^c^ Mean ± SD of three independent titration experiments.

### *In silico* dynamic and conformational effects of ATP binding

**Figure 5:**
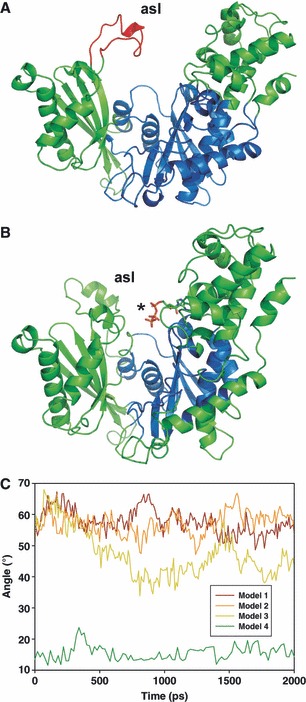
(A, B) Computed *B*-factor values and changes in the interdomain cleft angle. The computed *B*-factor values for the MD simulated model structures of the apoenzyme and the hGK–ATP binary complex. The values are colour-coded onto the 3D ribbon structure of (A) the apoenzyme and (B) the hGK–ATP binary complex, with red corresponding to the most mobile region (*B*-factor ≥ 400 Å^2^), blue corresponding to the most stable region (*B*-factor ≤ 40 Å^2^), and green corresponding to *B*-factor values in the range 40–400 Å^2^. Note also the change in secondary structure of the flexible active site loop (asl), comprising residues Ser151–Cys181, on binding of ATP (*). The *B*-factor values versus residue numbers are shown in Fig. S2B. (C) The changes in the interdomain cleft angle during the 2-ns MD simulations at 300 K. The change in the cleft angle was defined by the residues Lys169–Gly223 (‘hinge’)–Gly229, compatible with a partial closure of ∼ 15°. Model 1: hGK super-open conformation (including coordinates for the Glu157–Asn179 loop). Model 2: hGK super-open conformation with inserted Glc. Model 3: hGK super-open conformation with inserted ATP. Model 4: hGK ternary complex with Glc and ATP.

**Figure 6:**
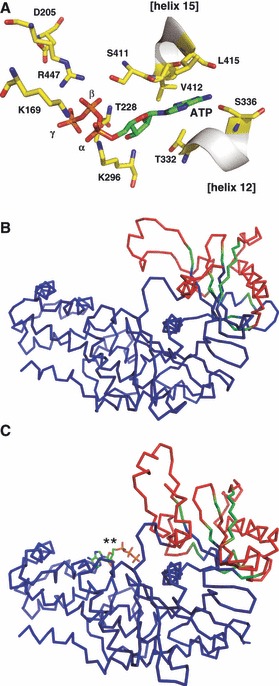
The ATP-binding site in the MD simulated model structure of the binary hGK–ATP complex and the domain motion induced by ATP binding to the hGK apoenzyme. (A) Close-up view of the ATP-binding site in the MD simulated model structure of the binary hGK-ATP complex, showing the main contact residues with ATP; for a presentation of all contact residues, see Table S1. For helix nomenclature, see [47]. (B, C) The domain motion induced by ATP binding to the apoenzyme with partial closure of the active site cleft and a rotation angle of ∼ 33°. The coordinates were those obtained for (B) the modelled super-open conformation, including the Glu157–Asn179 loop, and (C) the modelled open conformation with inserted ATP (GK–ATP). The C_α_ rmsd values were 4.01 Å for the whole protein, 2.09 Å for the fixed domain (349 residues), and 3.91 Å for the moving domain (87 residues). The dynamic domains were identified with the dyndom program [30]. The C_α_ backbone structures, shown in line presentation, were colour-coded as follows: blue, fixed domain; red, moving domain; and green, connecting residues. For comparison, the corresponding data for the domain motion induced by Glc binding to the apoenzyme are shown in Table S2. **ATP.

In the MD simulations, the starting crystal structure (PDB ID 1v4t) of the ligand-free super-open conformation was modified to include the 23 missing residues (Glu157–Asn179) in a surface loop structure (see Experimental procedures). The C_α_ rmsd value for the modelled structure and the crystal structure was ∼ 2.3 Å when the Glu157–Asn179 loop residues were not included. From the computed *B*-factor values (Figs 5A and S2B), the region that fluctuates the most is Glu157–Asn179, consistent with the observed disorder in the crystal structure. MD simulations of the modelled binary GK–ATP complex revealed that the global rmsd of the structure converged at the end of the 2-ns simulation period (Fig. S2A). The dynamic changes in the active site cleft opening over the 2-ns equilibration period (Fig. 5C), as defined by the residues Lys169–Gly223 (‘hinge’)–Gly229, suggest partial closure of the interdomain cleft (∼ 15°). These defining residues were previously used to monitor the opening of the cleft (∼ 50°) on MD simulations of Glc dissociation from the binary hGK–Glc complex [29]. A molecular motion was further indicated by the dyndom algorithm [30], with the coordinates obtained for the ligand-free form and the hGK–ATP complex at the end of the simulations (Figs 6B,C and S3; Table S2), also indicating partial closure of the cleft (∼ 33°) and an apparent domain motion, which were less dramatic than for the Glc-induced conformational transition (Table S2). In the final structure of the binary complex (Fig. 6A; Table S1), the adenosine moiety is packed between helices 12 and 15 in the L-domain [29] and stabilized by hydrogen bonds (with Thr332 and Ser336 in helix 12) and hydrophobic interactions (with Val412 and Leu415 in helix 15).

A conformational change was also indicated by the MD simulations of the modelled ternary hGK–Glc–ATP complex. In the final structure of the simulations, the interactions of the adenosine moiety were similar to those observed in the binary ATP complex, with the α-phosphate and β-phosphate oxygen atoms forming hydrogen bonds with Thr228 and Ser411 in the L-domain (data not shown).

For comparison, when the MD simulations were performed with Glc in the super-open conformation (Fig. 5C), no significant change in the interdomain cleft was observed. The substrate was found to be positioned at the active site, as expected [18], including the interactions with the primary contact residues Asn204 and Asn231 (data not shown). However, no interactions with Thr168 and Lys169 were seen, as the Ser151–Val181 surface loop was not displaced in the direction of Glc, and there was no measurable closure of the active site cleft during the 2-ns MD simulations (Fig. 5C), as observed in the crystal structures of the binary GK–Glc complex [18,31]. Thus, in this case, the simulation time (2 ns) was too short to demonstrate the large global conformational transition observed by ITF upon Glc binding, which has a millisecond to minute time scale [18,25,32], characteristic of this hysteretic enzyme, and thus out of reach of nanosecond-scale MD simulations.

### Steady-state kinetics

**Figure 7:**
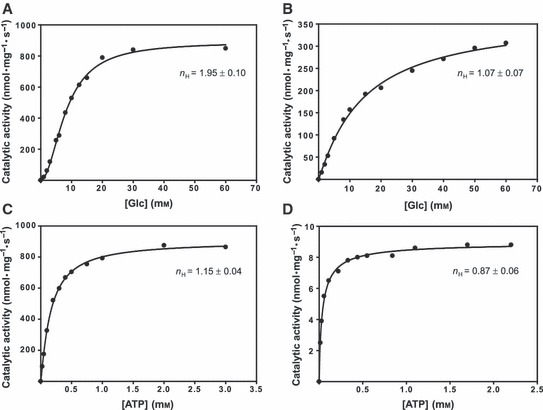
Steady-state kinetic properties of WT GST–hGK with Glc and MgATP as the variable substrates. (A) At 5 mm MgATP, positive cooperativity with respect to Glc was observed (*n*_H_ = 1.95 ± 0.10). (B) At a low (0.05 mm) concentration of MgATP, the cooperativity with respect to Glc was reduced (*n*_H_ = 1.07 ± 0.07). (C) At 60 mm Glc, the binding curve for MgATP was hyperbolic (*n*_H_ = 1.15 ± 0.04). (D) At a low (0.5 mm) concentration of Glc, negative cooperativity with respect to MgATP binding was observed (*n*_H_ = 0.87 ± 0.06). For all nonlinear regressions, the correlation coefficient (*r*^2^) was > 0.99. The steady-state kinetic constants are summarized in Table 3.

The steady-state kinetic properties of WT GST-hGK were determined with Glc as the variable substrate at high or low concentrations of MgATP (Table 3). Positive cooperativity was observed with respect to Glc at 5 mm (saturating) MgATP (*n*_H_ = 1.95 ± 0.10) (Fig. 7A) with an [S]_0.5_ value of 8.2 ± 0.3 mm. However, at 0.05 mm MgATP, the cooperativity was reduced to *n*_H_ = 1.07 ± 0.07 (Fig. 7B), and the [S]_0.5_ value was increased to 14.3 ± 1.7 mm. The fact that the kinetic cooperativity is dependent on the MgATP concentration is consistent with previous data reported for the rat liver isoform [33,34]. With MgATP as the variable substrate, a hyperbolic curve was obtained at a high Glc concentration (60 mm), with a *K*_m_ of 0.16 ± 0.01 mm (Table 3; Fig. 7C). However, at a low Glc concentration (0.5 mm), negative cooperativity was observed with respect to MgATP (*n*_H_ = 0.87 ± 0.06) (Fig. 7D), consistent with a previous report on the rat liver isoform [34], and the *K*_m_ was reduced to 0.04 ± 0.003 mm. Interestingly, the L146R mutant, with a severely reduced affinity for Glc (Table 1), demonstrated similar negative kinetic cooperativity with respect to MgATP as the variable substrate (*n*_H_ = 0.73 ± 0.04).

**Table 3:** The kinetic constants for WT GST–hGK at high and low concentrations of the fixed substrate. The catalytic activity was measured at 37 °C, as described in Experimental procedures. Kinetic parameters were calculated by nonlinear regression analyses with the Hill and Michaelis–Menten equations.

Conditions	Hill coefficient	[S]_0.5_ Glc (mm)	*K*_m_ MgATP^2−^ (mm)	*k*_cat_ (s^−1^)
Glc as variable substrate				
5 mm ATP	1.95 ± 0.10	8.23 ± 0.26	–	67.6 ± 1.3
0.05 mm ATP	1.07 ± 0.07	14.3 ± 1.7	–	27.8 ± 1.4
ATP as variable substrate				
60 mm Glc	1.15 ± 0.04	–	0.16 ± 0.01	68.4 ± 0.9
0.5 mm Glc	0.87 ± 0.06	–	0.04 ± 0.002	0.70 ± 0.01

## Discussion

The bisubstrate reaction catalysed by monomeric GK is mechanistically characterized by diffusion-controlled binding of Glc to thermodynamically favoured ligand-free conformations of the enzyme (Scheme 1), followed by global hysteretic isomerization of the enzyme to a closed conformation [29,31].

**Scheme 7:**
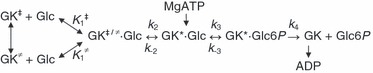
Reaction scheme for mammalian glucokinase.

From crystallographic, biophysical and kinetic studies on GK, it is known that both substrate binding and catalysis require substantial conformational changes in the enzyme. Ligand-free hGK is structurally dominated by a super-open conformation [31], which, in the crystal structure, is locked in an inactive state by electrostatic and hydrophobic interactions between the C-terminal helix (helix 17) and helix 6 [18]. Three residues (Asn204, Asn231, and Glu256) in the large domain [31] function as primary contact residues in the binding of Glc [18,31]. Pre-steady-state analyses of Glc binding to WT hGK [26,32,35] have provided evidence that the ligand-free enzyme in solution is in a pre-existing equilibrium between at least two conformers (marked as GK^‡^ and GK^≠^ in Scheme 1), i.e. the super-open conformation (∼ 80–95%) and an alternative (presumably less open) conformation (∼ 5–20%) with a higher affinity for Glc [26,35], which adds to the kinetic complexity of this reaction. Recent high-resolution NMR analyses and pre-steady-state Glc binding experiments also suggest that GK is capable of sampling multiple conformational states, both in the absence and the presence of Glc [32,36]. The global conformational changes triggered by Glc binding have been defined crystallographically [31]. In the closed conformation (marked as GK* in Scheme 1), precise alignment of additional substrate contact residues (notably Thr168 and Lys169 in the flexible surface/active site loop) [18,29] and the subsequent higher affinity for Glc efficiently accelerate the chemical reaction (*k*_3_) on binding of the cosubstrate MgATP. The overall binding constant *K*_1_ for Glc and the values for the forward (*k*_2_) and reverse (*k*_−2_) rates of the conformational transition, which probably includes intermediates [29,31,35,36], have been estimated by stopped-flow fluorescence spectroscopy [25]. In that study, the GK–Glc ↔ GK*–Glc interconversion was found to be slow, with *k*_2_ = 0.45 s^−1^ and *k*_−2_ = 0.28 s^−1^ (*K*_2_ = 1.6), favouring the forward rate and isomerization, whereas the isomerization was unfavourable with 2-deoxyglucose as the substrate (*K*_2_ = 0.8). Here, we present experimental evidence that ATP binds to the ligand-free form, and that this also results in changes in the protein conformation.

### ATP binds to the ligand-free open conformation of hGK

Previous attempts to demonstrate direct binding of ATP to the ligand-free form of rat GK by ITF spectroscopy [37] and hGK by differential scanning calorimetry [25] were reported to be unsuccessful. The topic was more recently readdressed [26] with a nonhydrolysable ATP analogue (AMP-PNP) and ITF, and relatively large quenching of the fluorescence signal was demonstrated, interpreted as a nucleotide-induced conformational change. However, as no corrections were made for a large inner filter effect, owing to the significant absorbance of the nucleotide at the excitation wavelength (285 nm), we have corrected for this effect here (at *λ*_ex_ = 295 nm) as well as for any effect of nonspecific binding to the enzyme (i.e. not in the active site), with the non-ATP-binding mutant form T228M (Table 1) as a reference enzyme. Our analyses revealed that AMP-PNP and ATP do indeed bind to hGK (Fig. 2) in the ligand-free open conformation, and the MD simulations (Fig. 6) further support this conclusion and also show the residues (including the mutated residue Thr228) directly contacting ATP at the active site of WT hGK. The partial quenching effect of AMP-PNP on the T228M reference enzyme with disrupted ATP binding at the active site (Fig. 2A,C) suggests a contribution of nonspecific binding of that nucleotide (i.e. not in the active site) in addition to its inner filter effect, as observed for free Trp. The idea that AMP, in contrast to MgADP^−^/MgATP^2−^, can bind to more than one site has been suggested for the rat liver isoform [16].

### Binding of ATP to ligand-free hGK results in a conformational change

High-resolution NMR analyses [36] have revealed that GK is an intrinsically mobile enzyme whose structure and dynamics are modulated by temperature and ligand binding. Here, we provide the first experimental evidence of ATP-dependent structural changes in WT hGK. Specifically, our ITF quenching (Fig. 2) and MD simulations (Figs 5 and 6) indicate a significant conformational change upon ATP binding, including motion of the flexible surface/active site loop and partial closure of the active site cleft (Figs 5C, 6B,C and S3). A change in conformation is further supported by the significant protective effect of ATP on binding of the extrinsic fluorescence probe ANS (Fig. 3A,B) and on the limited proteolysis by trypsin (Fig. 3C). In both assay systems, Glc showed more potent inhibition than ATP, which may be related to the larger conformational change and more effective closure of the active site cleft induced by Glc binding (Fig. S3; Table S2).

### Kinetic cooperativity with respect to Glc

In general, the mechanism for cooperativity observed in enzyme kinetic studies represents an experimental challenge. For monomeric GK, several models have been considered to explain the positive kinetic cooperativity with respect to Glc, including: (a) a random order mechanism of substrate (Glc and MgATP^2−^) addition [13,38]; and (b) a sequential order mechanism [15,39,40], in which the binding of Glc as the first substrate induces a slow, concentration-dependent conformational transition [34,40] characteristic of a hysteretic enzyme [41,42] (Scheme 1). The Glc-induced multiphasic ITF enhancement (millisecond to minute time scale) of WT and mutant forms of GK [18,25,26,35,37,43] strongly favours the second mechanism, and support the idea that the cooperativity can be explained by an equilibrium between conformational states with different affinities for Glc [18,25,26,35,37,43]. However, little experimental effort has been made to include or exclude any contribution of (Mg)ATP binding to the kinetic cooperativity.

The ligand-free and the binary GK–Glc complex are dynamic entities [32,36], and binding of (Mg)ATP may shift the equilibrium between different enzyme conformations, as shown for Glc [18,25,26,35,37,43]. In this study, the binding of ATP to the ligand-free enzyme was found, by four independent criteria, to trigger conformational changes, including partial closure of the active site cleft (Figs 5B, 6B,C and S3). Moreover, previous [34] and present (Table 1) steady-state kinetic analyses are also compatible with conformational control of GK catalytic activity by the binding of (Mg)ATP, with possible implications for kinetic cooperativity with respect to Glc. Thus, the cooperativity is largely reduced (*n*_H_ = 1.07 ± 0.07) at low concentrations of MgATP (Fig. 7B; Table 1) [34] and when MgATP is replaced by MgITP, a poor phosphoryl donor ATP analogue [44].

In most previous steady-state kinetic analyses, rat GK (liver) was observed to be noncooperative with respect to (Mg)ATP [33,45,46]. However, Neet *et al.* [34] reported negative kinetic cooperativity (*n*_H_ = 0.84) when measurements were made in the presence of 30% glycerol at a low Glc concentration (0.5 mm), and this was also observed here for the recombinant pancreatic hGK in the absence of glycerol (*n*_H_ = 0.87 ± 0.06). However, when hGK activity was measured at high glucose concentrations, the Hill coefficient for (Mg)ATP approached 1.0 (Fig. 7C), as expected from studies on rat liver GK [33,45,46]. Negative cooperativity (*n*_H_ = 0.73 ± 0.04) was also observed for the *GCK*-MODY mutant L146R, which has severely reduced affinity for Glc (Table 1). Moreover, our studies on this mutant revealed that the analogue ATPγS (at 2.5 mm) increases the mutant’s low equilibrium binding affinity for Glc (*K*_d_ decreases from 19.3 ± 3.8 mm to 14.0 ± 1.4 mm), as well as the Glc-induced fluorescence enhancement (by ∼ 25%) (Fig. 4). These effects may be related to partial catalytic activation following (Mg)ATP binding at physiological concentrations of Glc. Similar or possibly larger effects of ATP in promoting a catalytically competent state may occur in other mutations associated with *GCK-*MODY.

## Conclusions

Using biochemical and biophysical methods, we have obtained experimental evidence in support of binding of ATP to the ligand-free hGK, resulting in a change of protein conformation. The MD simulations indicate that the binding triggers molecular motion of the flexible surface/active site loop and partial closure of the interdomain active site cleft. The modelled structure of the hGK–ATP binary complex shows the residue contacts involved in ATP binding at the active site. Our findings further support conformational regulation of GK by ATP binding, with possible implications for kinetic cooperativity with respect to Glc. Further mutational studies, notably of *GCK*-MODY-associated mutations, may contribute to a better understanding of the mechanistic and functional implications of the multiple conformational equilibria and the conformational transitions induced by both Glc and (Mg)ATP, with possible future clinical implications.

## Experimental procedures

### Materials

The oligonucleotide primers used for site-directed mutagenesis were from Invitrogen (Carlsbad, CA, USA). The QuickChange XL Site-directed Mutagenesis Kit was from Stratagene (La Jolla, CA, USA). Glutathione Sepharose 4B was from Amersham Biosciences (GE Healthcare Europe GMBH, Oslo, Norway). Glc was from Calbiochem (San Diego, CA, USA). Magnesium chloride, magnesium acetate, guanidine hydrochloride, trypsin (bovine pancreas), trypsin inhibitor (soybean), pyruvate kinase (rabbit muscle), phospho(enol)pyruvate, ATP, ANS and AMP-PNP were from Sigma-Aldrich (St Louis, MO, USA). ATPγS was obtained from Roche Diagnostics Corporation (Indianapolis, IN, USA). All chemicals and buffers used for fluorescence measurements were of the highest analytical grade.

### Site-directed mutagenesis

The mutations T228M and L146R were introduced into the cDNA of the pancreatic (isoform 1) WT hGK with the QuikChange XL Site-directed Mutagenesis Kit. The pGEX-3X vector (kindly provided by F. M. Matschinsky, University of Pennsylvania, PA, USA), containing a protease factor Xa cleavage site, was used as the template. The following specific oligonucleotide primers were used for mutagenesis (mutated nucleotides are underlined): T228M forward, 5′-GC ATG ATC GTG GGC ATG GGC TGC AAT GCC TGC 3′; T228 reverse, 5′-GCA GGC ATTGCA GCC CAT GCC CAC GAT CAT GC-3′; L146R forward, 5′-CAC AAG AAG CTG CCC CGG GGC TTC ACC TTC TCC-3′; and L146R reverse, 5′-GGA GAA GGTGAA GCC CCG GGG CAG CTT CTT GTG-3′. Mutations were confirmed by DNA sequencing.

### Expression and purification of hGK

The WT and mutant recombinant proteins were generated and expressed in the form of GST fusion proteins in *Escherichia coli* BL21 cells, as previously described [47]. For the CD and guanidine hydrochloride unfolding experiments, the WT hGK was isolated by removing the GST fusion protein as previously described [18]. Purified protein was stored in liquid nitrogen in the absence of glucose (10 mm glutathione, 50 mm Tris/HCl, pH 8.0). The protein concentration was determined with the following absorption coefficients: *A*_280 nm_ (1 mg·mL^−1^·cm^−1^) = 1.05 (fusion protein); and *A*_280 nm_ = 0.65 (isolated protein) [18].

### Steady-state kinetics

The catalytic activity of GST–hGK was measured spectrophotometrically (*A*_340 nm_) at 37 °C by an NAD^+^-coupled assay with glucose-6-phosphate dehydrogenase, as previously described [18]. Kinetic studies were performed with 10–12 dilutions of Glc or ATP. The protocol with Glc as the variable substrate was carried out with 5 mm ATP (2.5 mm excess of Mg^2+^), whereas in the assay with MgATP as variable substrate (2.5 mm excess of Mg^2+^), saturating amounts of Glc were used. In the case of the severely inactivating mutation T228M [9,20], an ATP concentration of 10 mm was used. For determination of Hill coefficients of WT hGK, assays were performed with 12 different Glc concentrations (range: 0–60 mm) or with 11 or 12 concentrations of MgATP (range: 0–3 mm). In the assays with a low ATP concentration, an ATP-regenerating system was used, including 2 mm phospho(enol)pyruvate and 10 U of pyruvate kinase, to ensure a constant ATP level. Steady-state kinetic parameters were calculated by nonlinear regression analyses with the Hill and Michaelis–Menten equations. The reaction rates were measured from the linear part of the initial time-course.

### GST–hGK fluorescence measurements

ITF measurements were performed on a Perkin-Elmer LS-50B spectrometer (1-cm pathlength) at 25 °C in a buffer containing 20 mm Hepes, 100 mm NaCl, and 1 mm dithiothreitol (pH 7.0), and a protein concentration of 0.03 mg·mL^−1^ (a concentration of 0.04 mg·mL^−1^ was used for the mutant L146R GST–hGK). ATP titration experiments were performed in the same buffer containing 1 mm EDTA to complex trace amounts of Mg^2+^. The excitation and emission wavelengths used were 295 nm and 340 nm, respectively. Steady-state emission spectra were recorded from 305 nm to 500 nm, and slit widths for excitation and emission were set at 3 nm and 7 nm, respectively. All spectra represent an average of four scans. The binding isotherms and the apparent equilibrium dissociation constants (*K*_d_ for Glc and [L]_0.5_ for ATP) were determined by titration experiments; small aliquots of concentrated ligand (Glc/(Mg)ATP/AMP-PNP) were successively added to the enzyme solution every time that equilibrium was achieved after the previous addition (150–200 s). The change in intrinsic fluorescence intensity [(Δ*F*_eq_/*F*_0_) × 100] at *λ*_max_ (340 nm) was recorded as a function of the concentration of added ligand, with slit widths for excitation and emission set at 4 nm and 7 nm, respectively. The concentrations used were 0–60 mm Glc, 0–5 mm (Mg)ATP, and 0–450 μm AMP-PNP. To correct for the inner filter effect, caused by nucleotide absorbance at the excitation wavelength, the mutant T228M hGK, which is unable to bind ATP at the active site [9,20,21,24], was used as a reference enzyme. The specific WT hGK fluorescence response to added nucleotide was determined by subtracting the mutant T228M fluorescence response (inner filter effect) to added nucleotide. Titrations in the presence of free Trp (at a concentration giving the same *F*_0_ value as the enzyme) were performed as an additional control. The fluorescence intensity was adjusted for dilutions of protein, and baseline corrections were obtained with buffer without protein. Linear and nonlinear regression analyses of the data were performed as described previously [18], with sigmaplot technical graphing software (Systat Software, San Jose, CA, USA).

### ANS fluorescence measurements

Binding of ANS was performed at 38 °C on a Perkin-Elmer LS50B spectrometer (1-cm pathlength), with an excitation wavelength of 385 nm and excitation/emission slit widths of 6 nm. The cuvette, containing 60 μm ANS in 20 mm Hepes, 100 mm NaCl, and 1 mm dithiothreitol (pH 7.0), was pre-equilibrated to 38 °C before the reaction was started by the addition of hGK (0.75 μm). Five minutes later, the stable fluorescence emission spectra were recorded (400–600 nm). All spectra represent an average of four scans. When the effect of Glc (30 mm) or ATP (2 mm) on ANS binding was investigated, the ligand was added to the buffer in the cuvette and pre-equilibrated at the appropriate temperature before addition of enzyme. Prior to addition, the enzyme was preincubated for 7 min in the presence of the same concentration of ligand.

### CD spectroscopy

Far-UV CD spectra (185–260 nm, light path 1 mm) were recorded at 20 °C on a Jasco J-810 spectropolarimeter. The isolated WT and T228M proteins were diluted in 20 mm sodium phosphate buffer (pH 7.2) with 0.7 mm dithiothreitol to a final concentration of 23 μm. The proteins were analysed in the absence and presence of 40 mm Glc. The spectra obtained represent an average of four scans (scan rate of 50 nm·min^−1^), all background-corrected and smoothed. Secondary structure analyses were performed with the CD Neural Network algorithm [48]. Thermal unfolding (20–90 °C) was determined by following the change in ellipticity at 222 nm at a constant heating rate of 40 °C·h^−1^. The midpoint of the transition (*T*_m_) was determined from the first derivative of the smoothed denaturation curve. The associated high-voltage (pseudo-absorbance) increase was recorded at the same time as the CD data; this was found to be important for monitoring the quality and validity of the data (Fig. 1).

### Limited proteolysis with trypsin

WT GST–hGK was cleaved with factor Xa for 2 h at 4 °C, and the rate of limited proteolysis by trypsin was subsequently measured at 25 °C in a 100-μL reaction mixture containing 20 mm Hepes, 50 mm NaCl, and 2 mm dithiothreitol (pH 7.0), at a final GST–hGK concentration of 0.5 μg·μL^−1^ and a GST–hGK/trypsin ratio of 400 : 1 (by mass). The effect of preincubation with the substrates Glc (40 mm) or MgATP (2 mm ATP/4 mm MgAc) on the extent of proteolysis of WT hGK was studied. At timed intervals (0, 5, 10, 20 and 30 min), aliquots of 15 μL were taken from the proteolytic reaction, quenched with 14 μL of SDS sample buffer containing soybean trypsin inhibitor [protease/inhibitor ratio of 1 : 1.5 (by mass)], heated for 15 min at 56 °C, and subjected to 10% SDS/PAGE analyses. Bands were visualized by Coomassie Blue staining, and the band corresponding to full-length hGK was quantified with quantity one 1-d analysis software from Bio-Rad (Hercules, CA, USA). The data were fitted by nonlinear regression analysis with sigmaplot technical graphing software.

### Modelling of the hGK apoenzyme in the super-open conformation

The initial coordinates of the hGK apoenzyme were taken from the X-ray crystal structure solved at 3.4 Å resolution (PDB ID 1v4t) [31]. As the coordinates of a highly flexible loop structure, comprising residues Glu157–Asn179, were not fully resolved, we refined this crystal structure computationally. Initial coordinates for the 23 missing amino acids were taken from the structure of the binary glucose–hGK complex (PDB ID 1v4s) [including a GK activator (GKA) (compound A)] [31]. The dihedral angle of the peptide bond of Gly170 was adjusted so that the terminal amino acids were in an optimal position for filling the gap in the apoenzyme. Insertion of the missing residues was followed by energy minimization and 3-ns MD simulation (allowing only the inserted residues to move). The first 2 ns of simulation were performed in an implicit water environment (generalized Born model) to allow fast relaxation of the inserted peptide (avoiding friction with explicit water molecules). The resulting model was then solvated in explicit water in a periodic box, and simulated for another nanosecond to further relax the model.

### Structure modelling and MD simulations

The binary hGK–Glc complex (PDB ID 1v4s) [including a GKA (compound A)] was structurally aligned with the modelled structure of the complete hGK apoenzyme by applying the combinatorial extension method as implemented in the ce software [49]. Coordinates for glucose were extracted from the binary complex, and saved together with the hGK apoenzyme coordinates to construct an initial model of glucose bound to the super-open conformation. Furthermore, the coordinates of human hexokinase type I in complex with AMP-PNP (PDB ID 1qha) were aligned with our model to build the initial model for ATP-bound super-open hGK, and with the coordinates of the binary complex with Glc (PDB ID 1v4s) to generate starting coordinates for the ternary complex. These procedures provided us with the following four structural models: model 1, hGK super-open conformation (including coordinates for the Glu157–Asn179 loop); model 2, hGK super-open conformation with inserted Glc; model 3, hGK super-open conformation with inserted ATP; and model 4, hGK ternary complex with Glc and ATP.

Coordinates for all four models were relaxed by MD simulation in explicit water. The changes in rmsd values during the 2-ns MD simulations, relative to the starting structures, are given in Fig. S2A. Mg was not included in the initial simulation of the ternary complex.

### Software and hardware

MD simulations were performed with amber10 software [50]. The four models were all simulated in a truncated octahedral periodic box with TIP3P water, keeping bonds involving hydrogen atoms fixed, allowing a 2-fs time step between calculations of forces acting on the atoms. The temperature was increased from 0 K to 300 K under constant volume during the first 20 ps of the simulation, keeping the protein coordinates fixed with weak restraints. The systems were further equilibrated at 300 K for 200 ps before restraints were removed, and the models were allowed to relax for 2 ns under constant pressure and temperature. Coordinates were saved every 10th picosecond from the 2-ns simulations, and trajectories were analysed with the ambertools suite of programs (http://ambermd.org/#AmberTools). All simulations were performed on an HP BL 460c Linux cluster equipped with Xeon 2.66-GHz quad-core processors. Each job was distributed over 32 CPUs, and the average computation time was 11.5 h·ns^−1^.

By use of the crystal structure of the super-open (PDB ID 1v4t) and the closed (Glc and GKA-bound) (PDB ID 1v4s) forms, two structure-based methods were used for the estimation of the free energy of thermal unfolding [51] and the folding free energy [52] of hGK mutants.

The dyndom program [30] was used to determine dynamic domains and connecting residues involved in hinge bending motions on binding of Glc and ATP to the super-open conformation of hGK. Structural images were generated with pymol, version 1.0 [53]. icm-pro [54] was used to analyse the resulting complexes after MD simulations and to calculate the contact area of residues interacting with Glc and ATP presented in Table S1.

## Abbreviations

AdN: adenine nucleotide
AMP-PNP: adenosine-5′-(β,γ-imido)triphosphate
ANS: 8-anilinonaphthalene-1-sulfonate
ATPγS: adenosine-5′-*O*-(3-thiotriphosphate)
*GCK*-MODY: *GCK* maturity-onset diabetes of the young
GK: glucokinase
GKA: glucokinase activator
Glc: α-d-glucose
GST: glutathione-*S*-transferase
hGK: human glucokinase
ITF: intrinsic tryptophan fluorescence
MD: molecular dynamic
*n*_H_: Hill coefficient
PDB: Protein Data Bank
WT: wild-type

## Supporting Information

The following supplementary material is available:

**Fig. S1.** Far-UV CD spectra of WT and T228M hGK. **Fig. S2.** The atom-positional backbone rmsd ofthe MD trajectory structures during the 2-ns MD simulations,relative to the starting structures, and calculated *B*-factorvalues based on fluctuations of C_α_ carbons during the 2-ns MD simulations of the four modelled structures. **Fig. S3**. Surface presentations of theligand-free (open), ATP (partly closed) and Glc + ATP(closed) modelled structures of hGK. **Table S1.** Contact area in percentage of exposed area for residues interacting with Glc and ATP in the binary complex. **Table S2**. Domain motions in the conformationaltransitions induced by Glc and ATP binding to the apoenzyme.

Please note: As a service to our authors and readers, this journal provides supporting information supplied by the authors. Such materials are peer-reviewed and may be re-organized for online delivery, but are not copy-edited or typeset. Technical support issues arising from supporting information (other than missing files) should be addressed to the authors.
