# Supplementary material for: Binding of ATP at the active site of human pancreatic glucokinase – nucleotide-induced conformational changes with possible implications for its kinetic cooperativity
Source: FEBS J. 2011 May 31;278(13):2372–86. doi: 10.1111/j.1742-4658.2011.08160.x (PMC3531626; doi:10.1111/j.1742-4658.2011.08160.x)
Supplement: Supplementary file 1 — Fig. S1. Far-UV CD spectra of WT and T228M hGK. Fig. S2. The atom-positional backbone rmsd ofthe MD trajectory structures during the 2-ns MD simulations,relative to the starting structures, and calculated B-factorvalues based on fluctuations of Cα carbons during the 2-ns MD simulations of the four modelled structures. Fig. S3. Surface presentations of theligand-free (open), ATP (partly closed) and Glc + ATP(closed) modelled structures of hGK. Table S1. Contact area in percentage of exposed area for residues interacting with Glc and ATP in the binary complex. Table S2. Domain motions in the conformationaltransitions induced by Glc and ATP binding to the apoenzyme. [file febs0278-2372-sd1.zip › febs_8160_sm_FigS1-3&TableS1-2.pdf]

the FEBS Journal

www.febsjournal.org

Binding of ATP at the active site of human pancreatic glucokinase – nucleotide-induced conformational changes with possible implications for its kinetic cooperativity

Janne Molnes, Knut Teigen, Ingvild Aukrust, Lise Bjørkhaug, Oddmund Søvik, Torgeir Flatmark and Pål Rasmus Njølstad

DOI: 10.1111/j.1742-4658.2011.08160.x

SUPPORTING INFORMATION

Binding of ATP at the active site of human pancreatic glucokinase - nucleotide-induced conformational changes with possible implications for its kinetic cooperativity

Janne Molnes, Knut Teigen, Ingvild Aukrust, Lise Bjørkhaug, Oddmund Søvik, Torgeir Flatmark and Pål Rasmus Njølstad.

SUPPORTING FIGURES

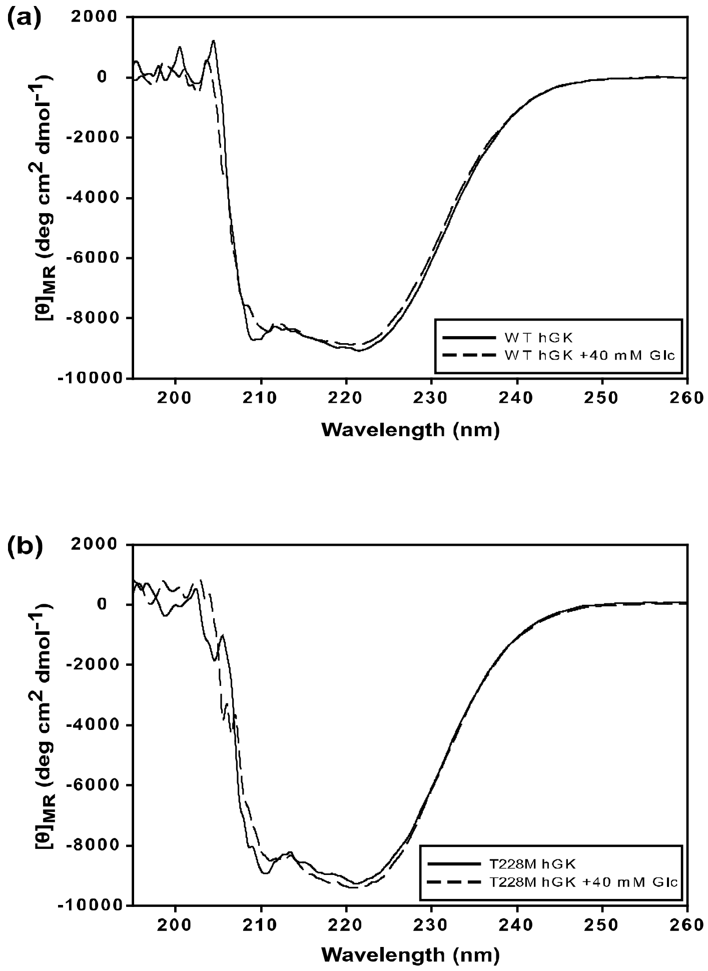

FIG. S1. Far-UV circular dichroism spectra of WT and T228M hGK. The spectra were obtained at 20 °C at a protein concentration of 23 μM in a 20 mM sodium phosphate buffer (pH 7.2) with 0.7 mM DTT in the absence and presence of 40 mM glucose for (a) WT and (b) T228M. The observed optical activity is expressed as the mean residue molar ellipticity $[\theta]_{MR}$ (deg cm^2 $dmol^{-1}$).

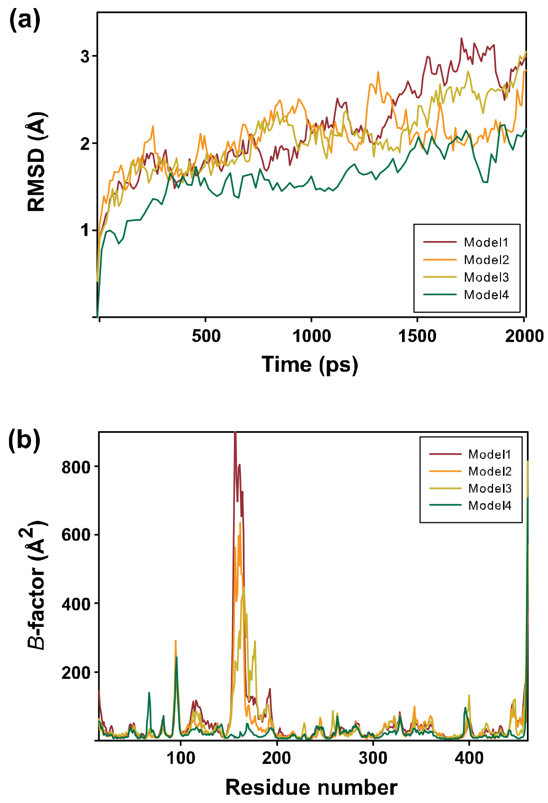

FIG. S2. (a) The atom-positional backbone rmsd of the MD trajectory structures during the 2 ns MD simulations, relative to the starting structures. (b) Calculated *B*-factor values based on fluctuations of C_α carbons during the 2 ns MD simulations of the four modelled structures.

MODEL 1: hGK super-open conformation (including coordinates for the E157-N179 loop).
MODEL 2: hGK super-open conformation with inserted Glc.
MODEL 3: hGK super-open conformation with inserted ATP.
MODEL 4: hGK ternary complex with Glc and ATP.

(a)

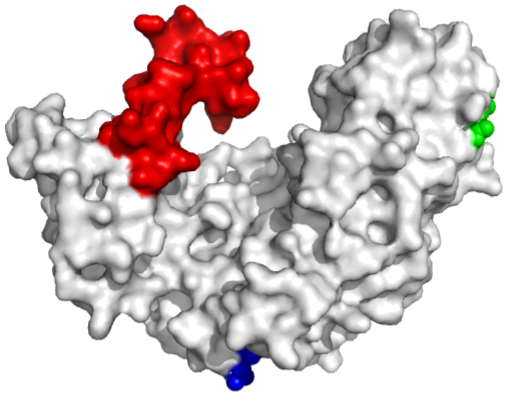

(b)

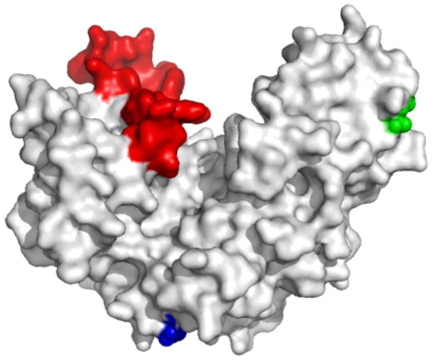

(c)

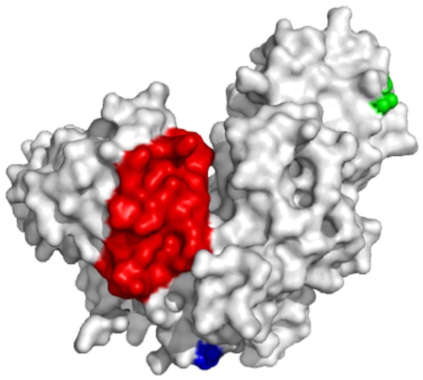

FIG. S3. Surface presentation of the (a) open unliganded hGK model structure, (b) the partly closed binary hGK·ATP complex and (c) the closed ternary hGK·Glc·ATP complex. The flexible active site loop (lid) is marked in red, the residue Q239 in blue and the residue E312 in green.

SUPPORTING TABLES

Table S1. Contact area in per cent of exposed area for residues interacting with α-D-Glucose and ATP in the binary complex

α-D-Glucose		Binary complex[a]
**Ligand**		**91**
T168	H-b	14
K169	H-b	16
N204	H-b	9
D205	H-b	21
N231	H-b	28
E256	H-b	11
S151		23
G229		21
E290		11
F152		8
Q287		5
C230		8
P153		4
T206		2
G258		2
**ATP**		**Binary complex[b]**
**Ligand**		**55**
T332		28
S336		20
S411		27
V412		19
L415		19
T228		21
K296		22
K169		10
R447		9
G295		35
R333		18
H416		8

[a]The crystal structure of the binary hGK·Glc complex (PDB i.d. 1v4s). It is not known to what extent the structure is perturbed by the simultaneous binding of a low-molecular-weight GK-activator (GKA).
[b]The MD simulated model structure of the binary hGK·ATP complex.
Abbreviation: H-b, hydrogen bond interaction.

Table S2. Domain motions in the conformational transitions induced by α-D-Glucose and ATP binding to the apoenzyme

Transition	rmsd (Å)			No. of residues in domains		Δ cleft angle (°)
	Whole prot.	Fixed dom.	Moving dom.	Fixed	Moving	
GK→GK·Glc[a]	9.51	2.91	6.10	306	104	~ 104 (closure)
GK→GK·ATP[b]	4.01	2.09	3.91	349	87	~ 33 (closure)

[a] The coordinates were PDB i.d. 1v4t, not including the E157-N179 loop residues (GK), and 1v4s (GK·Glc).
[b]The coordinates were the modelled super-open conformation including the E157-N179 loop (GK) and the modelled open conformation with inserted ATP (GK·ATP).
The dynamic domains and changes in interdomain cleft angles were determined by the DynDom program (Hayward and Berendsen 1998).

References

Hayward, S. and H. J. Berendsen (1998). "Systematic analysis of domain motions in proteins from conformational change: new results on citrate synthase and T4 lysozyme." Proteins **30**(2): 144-154.
